# Editorial: Molecular mechanisms in psychiatry 2023: anxiety and stress

**DOI:** 10.3389/fpsyt.2025.1632304

**Published:** 2025-08-13

**Authors:** Shinsuke Hidese, Francesco Rusconi, Dominik Strzelecki, Marco Grados

**Affiliations:** ^1^ Department of Psychiatry, School of Medicine, Teikyo University, Tokyo, Japan; ^2^ Department of Medical Biotechnology and Translational Medicine, Università degli Studi di Milano, Milan, Italy; ^3^ Department of Affective and Psychotic Disorders, Medical University of Lodz, Lodz, Poland; ^4^ Department of Psychiatry & Behavioral Sciences, Division of Child & Adolescent Psychiatry, Johns Hopkins University School of Medicine, Baltimore, MD, United States

**Keywords:** affective recognition, anxiety disorders, early chronic stress, pathomechanism, preclinical and clinical studies

The Research Topic “*Molecular Mechanisms in Psychiatry 2023: Anxiety and Stress*” intends to collect the most advanced research on the molecular mechanisms of anxiety and stress from a variety of disciplines across neurobiology and psychopharmacology. Based on the collected articles, we aimed to highlight novel directions for future research in the field of molecular mechanisms of anxiety and stress.

Anxiety disorders often have onset in youth, and their longitudinal course shows variable expression (1). The cellular-level pathophysiology of acute and chronic anxiety has considered the effect of circadian rhythm mechanisms, epigenetic expression, neurotransmitter differences, immune effects, and sex hormone levels (2). In turn, neuroimaging studies suggest identifiable neural circuit vulnerabilities that can lead to the emergence and maintenance of pathological anxiety. These vulnerabilities are further impacted by maladaptive behaviors that engage in the avoidance of extinction learning opportunities, thus prolonging anxiety symptom expression (3). Advancing current knowledge in the area of molecular mechanisms of anxiety and stress, Guo et al. have performed a bibliometric and visual analysis of the association between the pathogenesis of anxiety disorders and gut microbiota. The design identified 1,198 relevant articles, with an increase in interest in these topics being seen in the past two decades, with China, the United States, and Canada being the countries with the highest output. Key research themes included anxiety, gut microbiota, depression, stress, the gut–brain axis, and probiotics, providing a broad array of coverage for anxiety and stress.

Animal models have been used to illuminate the precise molecular mechanisms and brain circuitry differences associated with anxiety; an important use of these models is to provide a platform for the development of future anxiolytic drugs (4). In a preclinical study of this Research Topic, Yin et al. have reported on the effect of electroacupuncture and its anxiolytic effects using a mouse model of social isolation-induced anxiety. Using a standard test for anxiety and measuring stress responses (NADPH oxidase 2 (NOX2), microglial activation), electroacupuncture was shown to have an effect on mice, mitigating anxiety-like behaviors. At the same time, there was a reduction in NOX2 expression within the basolateral amygdala microglia, a reduction in reactive oxygen species, and a restoration of microglia morphology. Also using animal models, Mottarlini et al. used the communal nesting (CN) environment, a nest-sharing paradigm between several mothers of rat pups, and the early social isolation (ESI) setting to study its effects on pups. The CN environment increased prosocial behaviors in male pups, while ESI sensitized the glutamate synapse in the medial prefrontal cortex of male, but not female, rats. This sex-biased effect was pronounced at the molecular level, with concomitant NMDAR effects and recruitment of second messenger molecules occurring only in the male rats. The authors report that a CN environment contributes to shape social behavior and glutamate synapse homeostasis in the medial prefrontal cortex of ESI-exposed male rats but not in female rats. These studies advance the understanding of specific mechanistic approaches to the amelioration of anxiety in humans.

In clinical studies of this Research Topic, Shen et al. have reported the use of an affective flanker and flexibility tasks on 50 participants with generalized anxiety disorder (GAD). In those with GAD, poorer affective recognition abilities, accompanied by deficits in affective shifting, were observed in comparison to 50 healthy controls. In turn, Qian et al. have reported on the use of a subliminal affective facial recognition task, wherein a positive response tendency is observed in 34 participants with panic disorder in comparison to 43 healthy controls. The test was conducted after a course of cognitive-behavior therapy (CBT); participants with diminished anxiety after CBT also had a decreased false fear response bias. In an interesting longitudinal study, Jin et al. have reported on perceived anxiety and stress during childhood, which may increase the risk of mental illness, using the China Health and Retirement Longitudinal Study (CHARLS). The participants reported “tense or anxious” caregivers, and this was paired with their own risk of depression later on in life. Childhood exposure to caregiver anxiety and stress significantly increased the risk of depression in later life (p < 0.05), with stronger effects observed among individuals with female caregivers. Li et al. have reported on the use of friend leukemia virus integration 1 (FLI1) in 12 veterans with PTSD and 12 without PTSD. The authors found significantly increased FLI1 expression in peripheral blood mononuclear cells in those veterans with PTSD, compared to those without PTSD. In particular, CD4+ T cells were increased, with no notable changes in CD8+ T cells. With lipopolysaccharide (LPS) stimulation, there was a further increase in IL-6 and IFN-gamma in affected veterans. Microglia cells from PTSD-affected veterans also showed greater activation, suggesting that suppression of FLI1 may be a route to mitigate inflammation and microglial activation associated with PTSD.

In summary, the field of molecular mechanisms associated with the phenomenology of anxiety and stress needs to consider multiple streams of input. Consideration for adverse childhood experiences, allostatic load, hormonal influences, DNA methylation, inflammatory mechanisms, and aging-oxidative processes are all worthy of consideration (5). The Research Topic submissions highlight the importance of multiple determinants of anxiety and stress in humans which can then evolve into anxiety disorders.
